# Time series analysis of malaria cases to assess the impact of various interventions over the last three decades and forecasting malaria in India towards the 2030 elimination goals

**DOI:** 10.1186/s12936-024-04872-8

**Published:** 2024-02-15

**Authors:** Mrigendra P. Singh, Harsh Rajvanshi, Praveen K. Bharti, Anup R. Anvikar, Altaf A. Lal

**Affiliations:** 1 Foundation for Disease Elimination and Control of India, Mumbai, India; 2 Asia Pacific Leaders’ Malaria Alliance, Singapore, Singapore; 3 Indian Council of Medical Research–National Institute of Malaria Research, New Delhi, India; 4 Sun Pharmaceutical Industries Ltd, Mumbai, India; 5 Global Health and Pharmaceuticals, Inc, Atlanta, GA USA

**Keywords:** Time series forecasting malaria, Interrupted time series, Malaria elimination

## Abstract

**Background:**

Despite the progress made in this decade towards malaria elimination, it remains a significant public health concern in India and many other countries in South Asia and Asia Pacific region. Understanding the historical trends of malaria incidence in relation to various commodity and policy interventions and identifying the factors associated with its occurrence can inform future intervention strategies for malaria elimination goals.

**Methods:**

This study analysed historical malaria cases in India from 1990 to 2022 to assess the annual trends and the impact of key anti-malarial interventions on malaria incidence. Factors associated with malaria incidence were identified using univariate and multivariate linear regression analyses. Generalized linear, smoothing, autoregressive integrated moving averages (ARIMA) and Holt’s models were used to forecast malaria cases from 2023 to 2030.

**Results:**

The reported annual malaria cases in India during 1990–2000 were 2.38 million, which dropped to 0.73 million cases annually during 2011–2022. The overall reduction from 1990 (2,018,783) to 2022 (176,522) was 91%. The key interventions of the Enhanced Malaria Control Project (EMCP), Intensified Malaria Control Project (IMCP), use of bivalent rapid diagnostic tests (RDT-Pf/Pv), artemisinin-based combination therapy (ACT), and involvement of the Accredited Social Health Activists (ASHAs) as front-line workers were found to result in the decline of malaria significantly. The ARIMA and Holt’s models projected a continued decline in cases with the potential for reaching zero indigenous cases by 2027–2028. Important factors influencing malaria incidence included tribal population density, literacy rate, health infrastructure, and forested and hard-to-reach areas.

**Conclusions:**

Studies aimed at assessing the impact of major commodity and policy interventions on the incidence of disease and studies of disease forecasting will inform programmes and policymakers of steps needed during the last mile phase to achieve malaria elimination. It is proposed that these time series and disease forecasting studies should be performed periodically using granular (monthly) and meteorological data to validate predictions of prior studies and suggest any changes needed for elimination efforts at national and sub-national levels.

## Background

Malaria, a global public health issue, has been successfully eliminated from 41 countries through time-tested case management and vector control strategies. Despite these promising strides, in the year 2021, the World Health Organization (WHO) estimated an alarming 247 million malaria cases and 619,000 deaths worldwide. Of these cases, the WHO South East Asia (SEA) region accounted for about 2% of the global malaria burden. Amongst the SEA countries, India contributed approximately 79% of the malaria cases and 83% of the malaria-attributed deaths in the region [1].

Several countries in the WHO SEA region have eliminated malaria or are close to eliminating malaria. Maldives and Sri Lanka are malaria-free. Thailand, Timor-Leste, Bhutan and Nepal are referred to as “E2025” countries as they aim to achieve elimination by 2025. Countries of the Asia Pacific region have also committed to the malaria elimination goal of 2030, with the majority of cases concentrated in the island nations of Papua New Guinea and the Solomon Islands, with an Annual Parasitic Incidence of 65 and 119, respectively [1]. There is a common concern of emergence and spread of drug resistance from the Greater Mekong Sub region, which combined with the insecticide resistance poses a threat to realizing the goals of malaria elimination in the region.

The WHO Global Technical Strategy has set the objective to achieve at least 90% case reduction in the global malaria burden and zero indigenous malaria cases by 2030 [2]. Despite the commitment, not all countries are on track to meet the 2030 global target of reducing malaria case incidence and death rates by at least 90%. Certain countries in the region grapple with a complex interplay of socio-economic, ecological, and health system-related factors, which have proven formidable obstacles in their malaria elimination endeavours.

India’s malaria epidemiology is complex across diverse demography, topography, and socio-cultural landscapes, which presents a risk to sustain the reduction in malaria cases and achievements of the elimination goals in a timely manner [3]. The country has implemented the National Strategic Plan for Malaria Elimination 2017–22 [4] and the National Framework for Malaria Elimination (NFME) in 2016–2030 [3], describing the strategies of early diagnosis and prompt treatment, vector control, community engagement, and inter-sectorial cooperation, to achieve the national malaria elimination goal of 2030. The national programme has emphasized special focus on 27 high-priority districts with moderate to high malaria transmission.

Information obtained through disease forecasting and time series analysis of historical data presents an opportunity for the policymakers and programme managers to use curated intervention efforts in a context-specific manner for the high-burden areas that are posing problems for disease elimination. Several malaria forecasting studies have been conducted in India, China, Burundi, Mali, Afghanistan, Bhutan and Ethiopia [5–11]. However, the studies conducted in India either used hospital based passive malaria cases [12] or data from selected Indian states [13–15], which did not allow for the time series analysis and predictive malaria forecasting of the entire country.

The three inter-related objectives of this study were: (1) identification of the trends of malaria cases in India over the period 1990 to 2022 using time series analysis in order to forecast future malaria case burden for 2023 and 2030; (2) perform segmented regression on interrupted time series data to assess the impact of major interventions adapted in the national malaria programme over the period 1990 to 2022; and (3) conduct analysis to determine the association of extraneous independent factors with malaria incidence for the period of 2011 to 2021.

For forecasting analysis and assessment of the impact of commodity and policy interventions, country-wide malaria data over 3 decades was used. For analysis of predictors of malaria, the state-wise annual malaria data was used.

## Methods

### Study design

This ecologic study employed an explanatory time-trend design to study the annual trend of malaria cases during 1990–2022 and forecast future malaria cases, assessing the impact of major anti-malarial interventions during the study period and assessing the extraneous factors associated with malaria incidence in India.

## Data sources

Retrospectively reported annual malaria cases for the period of 1990 to 2022 was obtained from the World Malaria Reports (WMRs) 2012 and 2023. State-wise annual malaria incidence data for the period of 2011 to 2021 and the timelines of different interventions for malaria control in the country was obtained from the public domain of NCVBDC New Delhi [16]. The extraneous variables associated with malaria incidence were collected from Census of India, 2011; India State of Forest Report 2021, Forest Survey of India; Rural Health Statistics, National Health Mission, Government of India; Monthly rainfall data series for districts, states, and sub-divisions and all India, Additional Director General of Meteorology, Ministry of Earth and Science, India Meteorological Department [17–20].

## Estimation of trend and impact analysis of interventions

The annual trends of the time series data of malaria cases during 1990–2022 were plotted using centred moving averages, exponential, linear, and locally weighted regression lines. Over the last 3 decades, at different timepoints, several commodities and policy interventions have been introduced in India. Some of these interventions were novel, and some helped in strengthening and scaling up the existing strategies. The major interventions used in the present study were: (1) In 1997, the Enhanced Malaria Control Programme (EMCP) was operational in 181 selected tribal dominant districts of the country with the assistance of the World Bank. Under EMCP, case detection was improved with robust surveillance particularly in high endemic, hard to reach tribal dominated areas; (2) Further, in the year 2006, the World Bank-assisted Intensified Malaria Control Project (IMCP) was launched in inaccessible, high endemic and *Plasmodium falciparum* dominant areas of the country [21]. Emphasis was given to early diagnosis and prompt treatment; (3) On-spot diagnosis using monovalent rapid diagnostic test for *P. falciparum* (RDT-Pf) and artemisinin-based combination therapy (Artesunate + Sulfadoxine-Pyrimethamine (ACT-SP) as first-line treatment of *P. falciparum* introduced in 2005; (4) Accredited Social Health Activists (ASHAs) engaged to provide diagnosis and treatment of malaria cases at village level in 2009; (5) Bivalent RDTs for diagnosis of *P. falciparum* and *Plasmodium vivax* (RDT-Pf/Pv) infections and long lasting insecticidal nets (LLINs) for vector control in 2009 [22]; (6) Revised national drug policy with introduction of artemether-lumefantrine combination (ACT-AL) as first-line drug to treat confirmed *P. falciparum* cases in North Eastern states of the country in 2013; (7) Scaling up of LLINs coverage in malaria-endemic areas from 2015; and (8) Launch of NFME in 2016 [3, 4, 23].

The major change points have been identified in the years 1997, 2006, 2013, and 2016, to determine the impact of respective interventions using the Generalized Least Square (GLS) model along with autocorrelation via a corARMA function in R 4.3.2 (The R Project for Statistical Computing) to an Interrupted Time Series (ITS) malaria data by dividing into pre-intervention and post-intervention segment periods (equation below) [24]. Two dummy variables such as a binary indication of whether the intervention has taken place at the time_(x)_ and time elapsed since the intervention were created. The slopes of the interrupted time trends of malaria cases between these two segments were compared to the estimated quantum of change and their statistical significance. The immediate effect showed a decline in malaria cases during the year following the introduction of interventions along with the sustained effect showing a declining rate over the following years.

$$outcome=gls (malaria\, cases \sim time+intervention+postintervention\, time, data=xxx, method="\text{ML}", correlation =corARMA \left(p = n, q=n,form=\sim time\right))$$

## Prediction of malaria cases from 2023 to 2030

The data from WMR used in this study was from the years 1990 to 2022, which allowed for a robust non-seasonal disease forecasting analysis to predict future cases of malaria. The dataset was divided into two subsets: a training set and a testing set. The training set had been used to train the forecasting model, while the testing set was reserved to assess the model's performance on new and unseen data. The linear trend, quadratic, cubic, centred Moving Average (MA), LOWESS, Simple Exponential Smoothing (SES), Double Exponential Smoothing (ETS), Auto-correlation Integrated Moving Average (ARIMA), Holt’s additive and Holt’s multiplicative regression models were used for forecasting analysis.

Evaluating the accuracy of time series forecasting models is essential to ensure that the predictions are reliable and trustworthy. Measuring performance of time series prediction model provides capability of the forecast to the real values. The most common performance statistics used are: Mean Forecast Error (or Forecast Bias) indicates the tendency of a model to overestimate or underestimate where positive bias means the model tends to overestimate, while negative bias means it tends to underestimate; Mean Absolute Error (MAE) measures absolute average magnitude of error (difference between actual value and predicted value) in prediction without considering the direction; Mean Squared Error (MSE) measures the average of the squares of the errors, which gives more weight to the large errors; Root Mean Squared Error (RMSE) is the square root of the MSE which is easier to interpret. It provides an estimate of the standard deviation of the forecast errors; Mean Absolute Percentage Error (MAPE) measures the relative accuracy of a forecast by calculating the percentage difference between predicted and actual values; Mean Absolute Standard Error (MASE), Akaike Information Criterion (AIC) and Bayesian Information Criterion (BIC). The model accuracy was measured with the lowest Root Mean Square Error (RMSE), Akaike Information Criterion (AIC) and Bayesian Information Criterion (BIC), Mean Absolute Percent Error (MAPE), Mean Absolute Standard Error (MASE) in the time series data for the test period. Accurate forecasting using ARIMA requires normally distributed stationary data with constant mean and variance over time. The present study used annual time series data points due to the non-availability of seasonal malaria data. Therefore, a seasonality test was not performed. Augmented Dickey-Fuller test (ADF Test) was used to test whether a given time series is stationary or not. The regular differencing method was used to make stationary time series observations over the time period. In the differencing method, each observation was replaced by a difference between the current and previous observation [25]. The present study follows the EPIFORGE 2020 checklist for reporting forecasting and prediction research [26].

## Association of extraneous factors with malaria incidence

The univariate and multivariate generalized linear regression model was used to determine the extraneous independent factors associated with state-wise annual parasite incidence (API) data from 2011 to 2021 as the trend of malaria over the study period was roughly linear. Homoscedasticity test with residual plot, normality test with Q-Q plot and multi-collinearity test were also performed. The multi-collinearity effect between the independent variables included in the model was tested using a pairwise correlation matrix. The analysis did not reveal any strongly correlated variables. Hence, all the independent variables were included in the multivariate model, such as the proportion of the tribal population and literacy rate as per the 2011 Census (in the demography domain), vacant positions of ASHAs and peripheral health workers, number of health facilities (in the health infrastructure domain), and geographical areas under forest, hilly terrain and annual average rainfall (in geo-climatic domain).

The API is the number of malaria-positive cases per thousand population. Since API is highly dependent on the annual blood examination rate (ABER), it was standardized with the 10% constant ABER in the analysis. The multicollinearity between the independent variables was resolved before applying the multivariate linear regression model. All the statistical analyses were performed in R 4.3.2 (The R Project for Statistical Computing).

## Results

### Annual trends of malaria during 1990–2022

During 1990–2000, 2001–2010 and 2011–2022, 2.38 (95% CI 2.14, 2.63), 1.75 (95% CI 1.61, 1.89), and 0.73 (95% CI 0.45, 1.01) million malaria cases were reported annually, respectively. The annual percent decline was 0.64 (95% CI − 7.42, 8.69), − 2.15 (95% CI − 6.84, 2.54), and − 14.31 (95% CI − 27.67, − 0.95), respectively, during these 3 decades (Table 1). The per cent decline in malaria cases during the 3rd decade (2011–2022) was significantly higher than in 1990–2000 (coefficient = − 14.94; 95% CI − 27.95, − 1.94; p = 0.026); and 2001–2010 (coefficient = − 12.15; 95% CI − 25.16, − 0.99; p = 0.05).

**Table 1:** Trend of malaria during 3 decades of 1990–2000, 2001–2010, and 2011–2022

Species	Period	Yearly malaria cases (millions)						Yearly percent decline					
		Mean	95% CI		Median	Q1	Q3	Mean	95% CI		Median	Q1	Q3
			Lower	Upper					Lower	Upper			
Malaria	1990–2000	2.38	2.14	2.63	2.22	2.12	2.66	0.64	− 7.42	8.69	2.19	− 11.07	4.89
	2001–2010	1.75	1.61	1.89	1.80	1.56	1.87	− 2.15	− 6.84	2.54	1.34	− 5.16	2.45
	2011–2022	0.73	0.45	1.01	0.86	0.26	1.09	− 14.31	− 27.67	− 0.95	− 17.76	− 21.80	− 0.46
*P. falciparum*	1990–2000	1.00	0.90	1.09	1.01	0.88	1.14	4.02	− 4.78	12.82	1.38	− 4.60	16.15
	2001–2010	0.85	0.80	0.90	0.84	0.80	0.89	− 2.05	− 7.19	3.10	− 2.55	− 9.56	4.38
	2011–2022	0.42	0.25	0.59	0.49	0.14	0.68	− 12.51	− 29.80	4.78	− 17.40	− 24.22	− 5.48
*P. vivax*	1990–2000	1.38	1.19	1.58	1.27	1.19	1.65	− 1.57	− 11.47	8.34	− 0.92	− 10.95	8.41
	2001–2010	0.90	0.80	1.00	0.94	0.77	1.01	− 2.11	− 8.47	4.25	− 1.79	− 6.60	5.79
	2011–2022	0.30	0.19	0.42	0.34	0.13	0.40	− 14.75	− 27.86	− 1.64	− 15.92	− 20.13	− 6.45

CI: confidence interval; Q1: first quartile; Q3: third quartile

During the 3 decades i.e. 1990–2000, 2001–2010 and 2011–2022, the annual mean *P. falciparum* and *P. vivax* cases were 1.00, 0.85, 0.42 and 1.38, 0.90, 0.30 million, respectively. The annual percent decline during 3 decades was 4.02, − 2.05, and − 12.51 in *P. falciparum* and − 1.57, − 2.11 and − 14.75 in *P. vivax,* respectively (Table 1). The mean percent change during 3 decades in *P. falciparum* and *P. vivax* did not differ significantly (p > 0.05). Similarly, the difference in mean percent change between total malaria cases and two species (*P. falciparum* and *P. vivax*) was also not found significant statistically (p > 0.05).

**Fig. 1:**
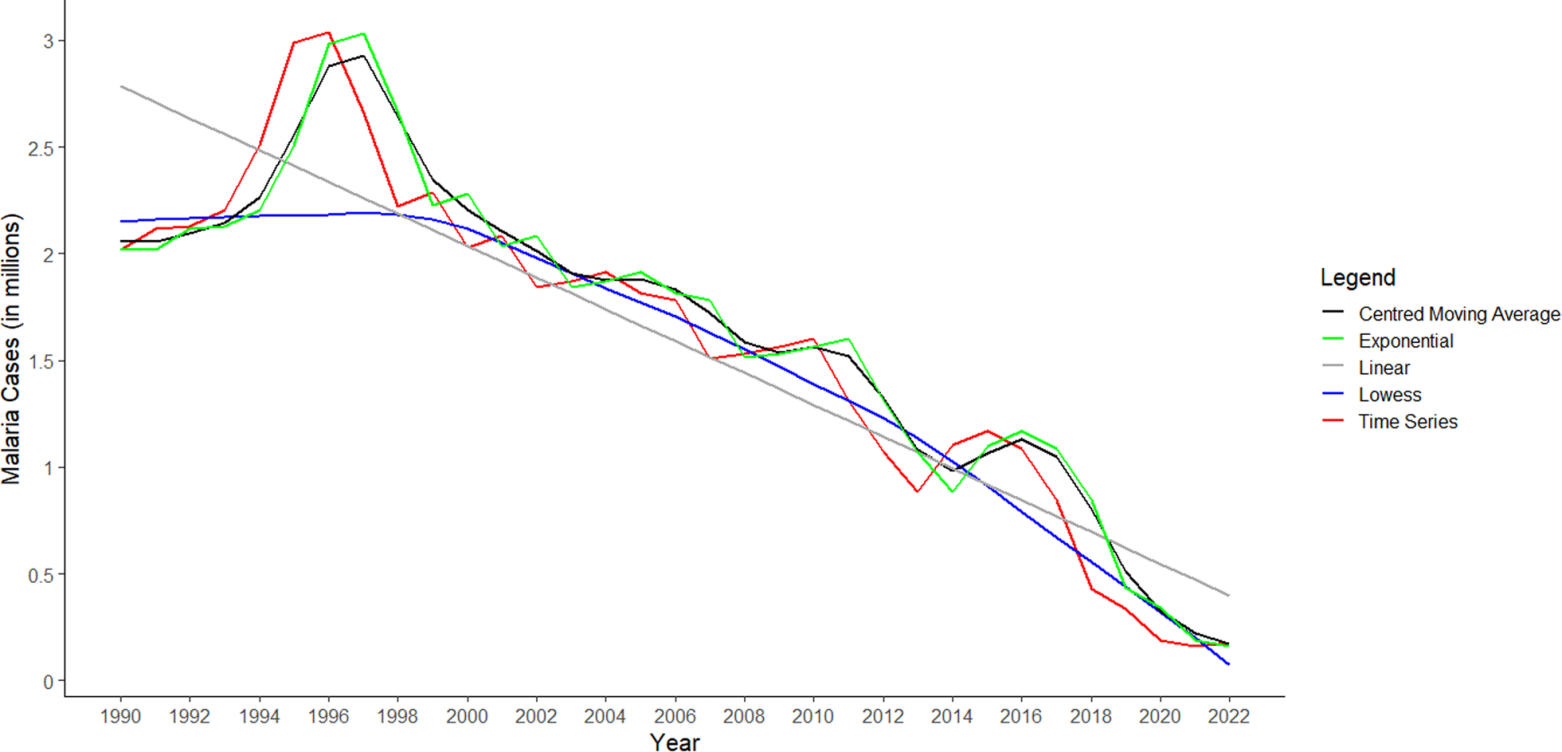
Trend of malaria cases in India from 1990 to 2022 showing time series (red line) and smoothing curves: (1) Centred moving average (black line); (2) Exponential curve (green line); (3) Linear curve (grey line); and (4) Lowess curve (blue line)

The temporal trend in malaria over the period of 1990 to 2022 revealed a 91.26% (95% CI − 91.29, − 91.22) reduction in the reported malaria cases. Overall, the average annual declining rate in the reported malaria cases was − 5.84% (95% CI − 11.56, − 0.12). Most of the annual decline was observed in 2017–2018 by 49%, which could be attributed to robust implementation of monitoring and evaluation frameworks of case management and vector control strategies that were part of the NFME 2016–30 (Fig. 1).

## Impact of major anti-malarial intervention from 1990–2022

**Fig. 2:**
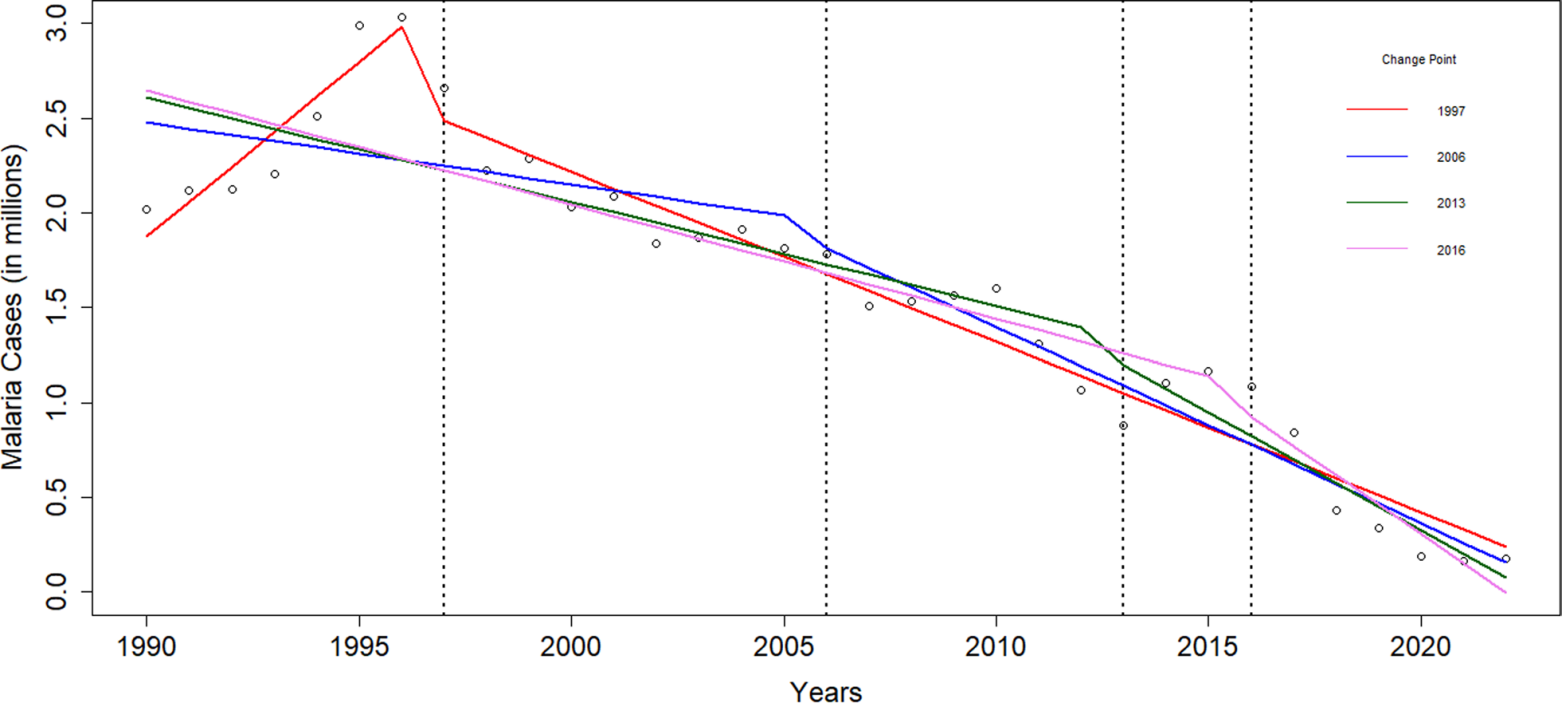
Segmented regression analysis in interrupted time series to assess the impact of major anti-malarial Interventions change point from 1990 to 2022. The four major change points are depicted in the graph. EMCP: enhance malaria control programme; IMCP: intensified malaria control programme; RDT: rapid diagnostic test; Pf: *Plasmodium falciparum*; Pv: *Plasmodium vivax*; ACT: artemisinin combination therapy; SP: sulfadoxine pyrimethamine; ASHA: accredited social health activist; AL: artemether lumefantrine; NFME: national framework of malaria elimination; LLIN: long lasting insecticidal nets

The analysis of ITS has revealed that EMCP, which focused on tribal-dominated hilly forests and *P. falciparum*-prone areas, had the most significant impact and effects sustained during the post-intervention period (p < 0.0001). IMCP provided early case detection and prompt treatment using RDT-Pf and ACT-SP, which contributed to a significant yearly cases reduction of malaria (p = 0.002). ASHAs (since 2009) and diagnosis using RDT-Pf/Pv (since 2013), and treatment with ACT-AL (since 2013) in the North Eastern region states led to an additional reduction in malaria cases, which was significant (p = 0.048). The NFME and scaling-up of LLINs distribution in hard-to-reach high malaria endemic areas had the highest impact in the reduction of malaria cases. However, in the GLS regression model, the coefficient was not found statistically significant (p = 0.101), possibly due to the fewer follow-up years after the change point (Table 2; Fig. 2).

**Table 2:** Interrupted time series segmented regression analysis showing the impact of major anti-malarial interventions from 1990 to 2022

Year of change point	Interventions*	Factors	Beta coefficient	Standard error	t	P value
1997	EMCP	Intercept	− 366,103,779	63,332,205	− 5.781	< 0.0001
		Year	184,914	31,777	5.819	< 0.0001
		Immediate effect	− 406,760	133,185	− 3.054	0.005
		Sustained effect	− 274,754	32,080	− 8.565	< 0.0001
2006	IMCP + RDT(PF) + ACT (ASP)	Intercept	67,536,474	30,212,557	2.235	0.033
		Year	− 32,693	15,125	− 2.161	0.039
		Immediate effect	− 70,923	194,286	− 0.365	0.718
		Sustained effect	− 70,867	20,480	− 3.460	0.002
2013	ASHA + RDT(PF + PV) + ACT(ASP/AL)	Intercept	112,158,649	18,390,013	6.099	< 0.0001
		Year	− 55,050	9190	− 5.990	< 0.0001
		Immediate effect	− 77,516	232,003	− 0.334	0.741
		Sustained effect	− 69,221	33,475	− 2.068	0.048
2016	NFME + RDT(PF + PV) + ACT(ASP/AL) + LLIN (scale-up)	Intercept	122,813,847	15,355,727	7.998	< 0.0001
		Year	− 60,384	7668	− 7.875	< 0.0001
		Immediate effect	− 58,668	271,886	− 0.216	0.831
		Sustained effect	− 94,662	55,948	− 1.692	0.101

^*^EMCP: enhance malaria control programme; IMCP: intensified malaria control programme; RDT: rapid diagnostic test; Pf: plasmodium falciparum; Pv: plasmodium vivax; ACT: artemisinin combination therapy; SP: sulfadoxine pyrimethamine; ASHA: accredited social health activist; AL: artemether lumefantrine; NFME: national framework of malaria elimination; LLIN: long lasting insecticidal nets

## Prediction of malaria from 2023 to 2030

**Fig. 3:**
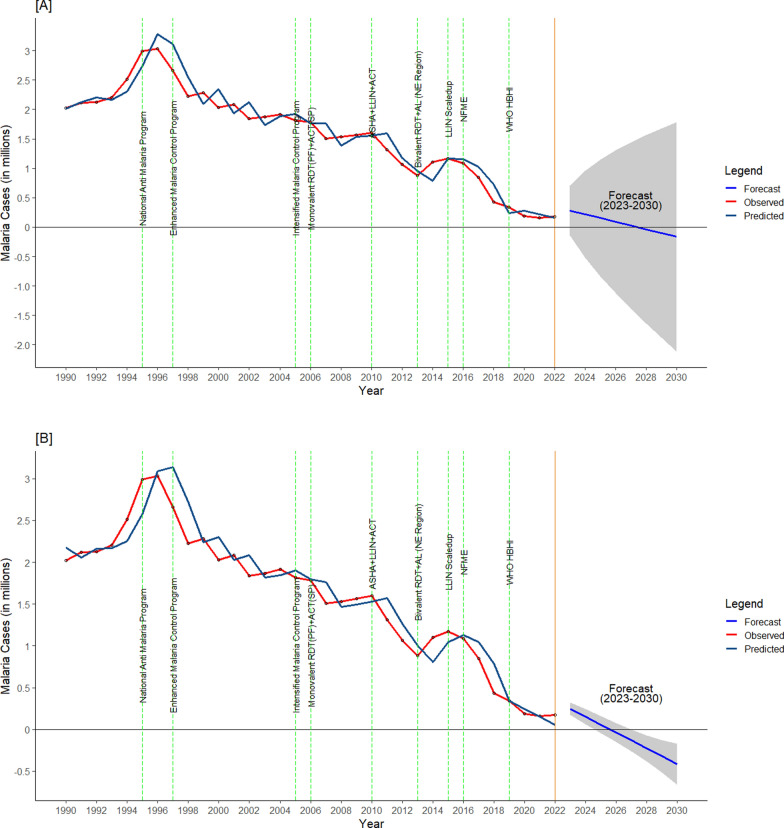
Forecast analysis using time series data from 1990 to 2022 and prediction of malaria cases from 2023 to 2030 using (**A**) ARIMA and **B** Holt’s additive models. The various interventions from 1990 to 2022 are depicted in both models

The statistical accuracy was found highest in ARIMA (1,2,2) model, with the lowest RMSE (64,631), AIC (774.15), BIC (779.48), MAPE (32.57), and MASE (0.34) indicator scores. The next lowest values of these parameters were in Holt’s additive and followed by Holt’s multiplicative models (Table 3). The MASE in Holt’s multiplicative model is more than 50% (0.51) which showed it to be an inappropriate model of forecasting for the present time series data. However, the 95% confidence interval in the predicted malaria cases was wider in ARIMA (1,2,2), as compared to both Holt’s models. The forecasting of the malaria cases for the next eight years (2023–2030) showed that the rapid decline from 2017–2022 was likely to continue, assuming the extraneous factors would be constant and there are no outbreaks. These observations imply that the target level of zero indigenous malaria cases would be achieved by 2027–2028 (Fig. 3).

**Table 3:** Measuring the accuracy of ten prediction models using nine summary statistics criteria

Models (n = 10)		Summary statistics (n = 9)								
		ME	RMSE	MAE	MPE	MAPE	MASE	ACF1	AIC	BIC
Linear	Training	− 2.71e−11	305,647.6	224,512.4	− 6.74	17.31	1.34	0.65		
	Test	− 4.33e + 05	436,429.1	433,277.9	− 248.19	248.19	2.59			
Quadratic	Training	2.72e− 11	250,728.0	189,867.5	− 1.92	11.90	1.13	0.66		
	Test	8.41e + 04	148,494.2	122,609.9	49.43	70.07	0.73			
Cubic	Training	− 2.52e− 11	262,603.7	191,917.9	− 2.08	11.82	1.15	0.67		
	Test	1.38e + 05	197,841.6	155,947.6	80.50	90.17	0.93			
Moving average	Training	− 70,280.17	183,319.75	145,459.7	− 10.08	13.57	1.05	0.51		
	Test	5797.66	11,713.42	10,721.0	2.98	6.02	0.08			
Loess	Training	− 7111.25	47,878.78	21,823.28	− 0.65	1.34	0.32	− 0.12		
	Test	342,636.37	355,731.07	342,636.37	197.43	197.43	5.06			
Simple Exponential	Training	− 67,907	216,932	164,871	− 10.29	15.6	1.05	0.25		
	Test	− 2,009,565	2,015,929	209,565	− 1147.84	1147.84			932.35	936.84
Double Exponential	Training	− 68,756	216,914	164,812	− 10.56	15.36	0.97	0.23		
	Test	− 2,113,922	2,122,632	2,113,922	− 1213.69	1213.69			932.34	936.83
ARIMA(1,2,2)	Training	− 47,088	197,391	157,307	− 3.96	11.62	0.94	− 0.06		
	Test	− 43,021	64,631	57,451	− 24.39	32.57	0.34		774.15	779.48
Holt's additive	Training	− 54,609	213,525	162,359	− 5.72	11.74	0.97	0.24		
	Test	24,779	77,470	63,311	14.82	35.48	0.38		852.06	857.67
Holt's multiplicative	Training	− 42,161	225,610	172,941	− 6.12	13.15	1.03	0.35		
	Test	− 86,303	90,667	86,303	− 49.43	49.43	0.51		855.31	860.92

ME: mean error; RMSE: root mean square error; MAE: mean absolute error; MPE: mean percent error; MAPE: mean absolute percent error; MASE: mean absolute scaled error; ACF: auto correlation function; AIC: akaike information criterion; BIC: bayesian information criterion

## Factors associated with malaria incidence

Univariate and multivariate linear regression analysis revealed that populations classified under Scheduled Tribes (indigenous people in India) were positively associated, and literacy rate was inversely associated with the API, both of which were statistically significant (p ≤ 0.001). Health infrastructure, which included the vacant position of ASHA workers (community-level health provider) per 1000 population and the vacant position of health workers at peripheral health facilities such as sub-centres, primary health centres, community health centres, and district hospitals (facility-level health provider) per 1000 population was positively associated with API (p ≤ 0.001). Whereas the number of health facilities per square km of geographical area was inversely associated with API (p = 0.04). Further analysis revealed that geographical areas under forest cover, hilly terrain, and annual average rainfall (mm) were positively associated with API (p < 0.0001). In the multivariate model, 37% of the variance in the API was attributed to the above independent factors included in the analysis (Table 4).

**Table 4:** Regression analysis of factors associated with the Annual Parasite Incidence (standardized with annual blood examination rate**)**

FACTORS	Univariate		Multivariate (R^2^ = 0.37)	
	Beta coefficient (95% CI)	P value	Beta coefficient (95% CI)	P value
*Demographics*				
Scheduled Tribe (%)	0.04 (0.03, 0.05)	< 0.0001	0.02 (0.01, 0.03)	0.001
Literacy (%)	− 0.10 (− 0.13, − 0.08)	< 0.0001	− 0.09 (− 0.012, − 0.07)	< 0.0001
*Health infrastructure*				
Proportion of vacant position of ASHA worker/000 pop	10.98 (8.65, 13.30)	< 0.0001	5.75 (2.27, 9.24)	0.001
Proportion of vacant position of health worker*/000 pop	7.73 (6.84, 8.61)	< 0.0001	8.95 (7.26, 10.63)	< 0.0001
Number of Health Facilities (SC, PHC, CHC, SDH, DH)/sq km	− 3.87 (− 5.22, − 2.51)	< 0.0001	− 1.46 (− 2.85, − 0.06)	0.04
*Geography*				
Forest covered (%)	0.04 (0.03, 0.05)	< 0.0001	0.02 (0.01, 0.03)	< 0.0001
% of Hilly Districts	0.01 (0.05, 0.01)	< 0.0001	0.02 (0.02, 0.03)	< 0.0001
*Climate*				
Annual Rainfall mm	0.0004 (0.0001, 0.0007)	0.002	0.0005 (0.0003, 0.0008)	< 0.0001
Constant			5.54 (3.88, 7.20)	< 0.0001

## Discussion

The use of longitudinal historical data for assessing the impact of intervention and forecasting studies is crucial for identifying interventions that have the most impact on achieving malaria elimination goals at national and sub-national levels. For conducting malaria forecasting analysis, the data quantity, quality, timelines, and consistency in reporting are key requirements. Generalized linear, ARIMA, and Holt–Winter’s models are the most commonly used statistical models in malaria forecasting studies. The selection of the appropriate forecasting model depends on the predictive accuracy, which is largely determined by RMSE, MAPE, MASE, MAE, MAD, 95% confidence intervals, and visual observation. In addition, AIC and BIC are also used as model-fitting criteria [12, 27, 28].

This study has used reported malaria cases data from the WMRs instead of the controversial estimated cases data. In India, efforts are being made to bridge the gap between reported and estimated cases with the increased community level awareness about the significant value of accessing public health systems. The mandatory reporting of malaria cases data by the private sector in almost all states of the country in the last 18 months and the introduction of newer data reporting tools such as the Integrated Health Information Platform are expected to shrink this gap.

The experiences from the Malaria Elimination Demonstration Project (MEDP) conducted for four years in Mandla, Madhya Pradesh have confirmed that treatments that are home-based or use alternate systems of medicine do not add significantly to the case data of state national programs. Through active surveillance and RDT-based diagnostics, it was found that malaria-attributable fever never went beyond 1%, so the individuals who receive treatment at home or receive treatment through alternative systems will not add significantly to the malaria case burden [29].

The present study has revealed a linear declining trend of malaria cases in India, with about a 91% reduction from 1990 to 2022. Between 1990 and 2000, there was an approximately 0.64% yearly increase in malaria cases. From 2001 to 2010, there was an annual malaria case reduction of about 2%, which could be attributed to the combination of several policy and commodities interventions such as the introduction of monovalent RDTs, replacement of chloroquine with ACT-SP for *P. falciparum* infections, enhanced surveillance under the IMCP, introduction of LLINs, and ASHAs. Complimenting the interventions done during the above period, in 2011–2022, the addition of NFME, bivalent RDTs, AL, and scaling-up of LLINs further contributed to about 14% yearly decline in malaria cases.

EMCP had the highest impact, possibly because in the year 1996, the number of malaria cases was higher. The impact of EMCP was due to a number of interventions introduced through EMCP, particularly in tribal-dominated high-burden areas of the country. RDTs for *P. falciparum* in the national programme. While the terms EMCP or IMCP are not mentioned now, but the components of the intervention have been continued with the addition of various new tools and methodologies.

ARIMA and Holt’s time series regression models are the most common tools for disease forecasting [30]. In the present study, the ARIMA (1,2,2) was the most fitted model to predict malaria cases compared to nine other models. The ARIMA (1,2,2) model predicted that zero malaria cases might be achieved in the year 2028. However, there was a wide range of 95% Confidence Interval (CI). Further, Holt’s additive model predicted the achievement of zero malaria cases by the year 2026 to 2028. In a state-wide study done in Odisha, the authors found that Holt’s Winter was the most fitted model across varying endemicities, which predicted a slowing down of the decline in 2014–2016, hence, missing the state elimination goal [13].

The present study has found that the vacant positions of health providers at the community and facility levels were associated with an increased risk of malaria incidence. It has also revealed that if the health care facilities per square kilometre were to be increased, particularly in hard-to-reach malaria high-endemic areas, the incidence of malaria could decrease. However, while assessing the relationship between healthcare facilities and malaria incidence in remote areas, studies have found that increased travel time and distance to healthcare facilities significantly affect the likelihood of seeking care. For instance, a study in Uganda indicated that as travel time to a health facility increased, the probability of seeking care for malaria symptoms decreased [31]. Circular associations may arise if, for example, high malaria incidence deters healthcare professionals from working in these areas, further exacerbating staff shortages and weakening the healthcare infrastructure. Consequently, this can lead to a vicious cycle where increasing malaria incidence and decreasing healthcare provision could reinforce each other.

The present study has also found that tribal-dominated, hilly and forested areas have a greater risk of malaria infection. This finding is complemented by a study done in the state of Madhya Pradesh, where it was found that communities with high literacy had a lower burden of malaria, possibly because of better health-seeking behaviour [32]. The phenomenon of high literacy and low malaria burden is supported by the national trends where most malaria burden is found in inaccessible hilly forested terrains and tribal-dominated areas with poor healthcare infrastructure and low levels of literacy [33].

The Goalkeepers Report 2021 by the Bill and Melinda Gates Foundation (BMGF) has predicted that the global malaria cases would be 32 new cases per 1000 people in 2030, which is almost the same as the reported malaria burden of 31 new cases per 1000 people in 2020. The report gives a range of 21–42 cases per 1000 population as the best and the worst situations in 2030, respectively. A similar trend has been predicted for South East Asia + East Asia + Oceania, predicting zero to one case per 1000 population as the best and the worst situations in 2030, respectively. In comparison, in sub-Saharan Africa, 68 to 195 malaria cases per 1000 population are predicted as the best and worst-case scenarios in 2030. For India, the prediction showed one to four cases per 1000 population in 2030, indicating a potential miss of the national malaria elimination goal [34]. However, the present study has suggested that India might be able to achieve the national malaria elimination goal, subject to the absence of disease outbreaks, climatic changes, emergence of anti-malarial drug resistance, and other independent factors. It is also critical to use context specific and curated elimination protocols for each of the 27 high burden districts in India to eliminate indigenous transmission. If this is accomplished in three to four years, as was demonstrated in Mandla through MEDP, and there are no disease outbreaks and issue related to climate change and drug resistance a malaria free India in 2030 is possible.

A study conducted in Odisha state reported about three times higher annual declining rates in malaria incidence during the intensified post-intervention period (2009–2013) compared to the pre-intervention period (2003–2008). This study attributed the drop to integrated vector control measures, rapid diagnosis and prompt treatment, service decentralisation, inter-sectoral convergence, and behaviour change communication (13). LLINs, RDTs with ACT and biological vector control interventions helped in a significant reduction in malaria cases during 2011–2021 in the Karnataka state [35]. The present study has also found that optimal coverage of ITNs/LLINs effectively decreased the malaria caseload. Similarly, ITNs/LLINs were found to be a cost-effective intervention tool in Bangladesh [36] and Sri Lanka, which have been malaria-free since 2016 [37].

This study used reported annual malaria cases in the absence of granular and monthly malaria data of the country in public domain. Therefore, seasonality and geographical variability in malaria cases could not be analysed in the time series modelling. Further, in the present forecasting model, the effect of climatic variables such as rainfall, humidity, and temperature have not been quantitated, although these are significant covariates of malaria as reported by other studies [38]. The data in the context of roads and telecommunications infrastructure were also not analysed in the present study.

## Conclusion

The significant decrease in malaria incidence in India from 1990 to 2022 highlights the successful implementation of various anti-malarial strategies and interventions. This study reveals a significant negative trend in malaria cases over the past 3 decades, with a remarkable reduction of 91%. Factors contributing to this substantial decrease include focused interventions such as EMCP, IMCP, ASHAs’ contribution, RDT-Pf/Pv and ACT deployment, the NFME, and scaling-up of LLINs distribution.

Out of these predictive models, the ARIMA and Holt’s additive have shown reliable predictive capabilities, indicating that the decreasing malaria cases are likely to continue, forecasting zero indigenous malaria cases by 2027–2028. The study also identified the most impactful combination of intervention packages. Disease modelling studies will have the most impact during the last mile of disease elimination, provided the information is used in real-time by the programmes and policymakers. It is proposed that such time series and disease modelling efforts should be repeated periodically to validate prior predictions of recent years and suggest any changes needed in the interventions required for a malaria-free India.

## Data Availability

Data analysed in the present study was obtained from public domain and the references have been cited. The analysed data is available with corresponding author.
